# The value of Augmented Reality in surgery — A usability study on laparoscopic liver surgery

**DOI:** 10.1016/j.media.2023.102943

**Published:** 2023-12

**Authors:** João Ramalhinho, Soojeong Yoo, Thomas Dowrick, Bongjin Koo, Murali Somasundaram, Kurinchi Gurusamy, David J. Hawkes, Brian Davidson, Ann Blandford, Matthew J. Clarkson

**Affiliations:** a Wellcome ESPRC Centre for Interventional and Surgical Sciences, University College London, London, United Kingdom; b UCL Interaction Centre, University College London, London, United Kingdom; c Division of Surgery and Interventional Sciences, University College London, London, United Kingdom

**Keywords:** Augmented reality, Image-guided surgery, Usability, Laparoscopic surgery

## Abstract

Augmented Reality (AR) is considered to be a promising technology for the guidance of laparoscopic liver surgery. By overlaying pre-operative 3D information of the liver and internal blood vessels on the laparoscopic view, surgeons can better understand the location of critical structures. In an effort to enable AR, several authors have focused on the development of methods to obtain an accurate alignment between the laparoscopic video image and the pre-operative 3D data of the liver, without assessing the benefit that the resulting overlay can provide during surgery. In this paper, we present a study that aims to assess quantitatively and qualitatively the value of an AR overlay in laparoscopic surgery during a simulated surgical task on a phantom setup. We design a study where participants are asked to physically localise pre-operative tumours in a liver phantom using three image guidance conditions — a baseline condition without any image guidance, a condition where the 3D surfaces of the liver are aligned to the video and displayed on a black background, and a condition where video see-through AR is displayed on the laparoscopic video. Using data collected from a cohort of 24 participants which include 12 surgeons, we observe that compared to the baseline, AR decreases the median localisation error of surgeons on non-peripheral targets from 25.8 mm to 9.2 mm. Using subjective feedback, we also identify that AR introduces usability improvements in the surgical task and increases the perceived confidence of the users. Between the two tested displays, the majority of participants preferred to use the AR overlay instead of navigated view of the 3D surfaces on a separate screen. We conclude that AR has the potential to improve performance and decision making in laparoscopic surgery, and that improvements in overlay alignment accuracy and depth perception should be pursued in the future.

## Highlights

• Study to quantify benefit of Augmented Reality in Laparoscopic surgery.
• Phantom setup designed for participants to intra-operatively locate targets.
• Three Image Guidance scenarios tested with both surgeons and non-surgeons.
• Accuracy, completion time and usability scales show value of Augmented Reality.
• Further research should consider alignment accuracy and depth perception.

## Graphical abstract

**Figure:**
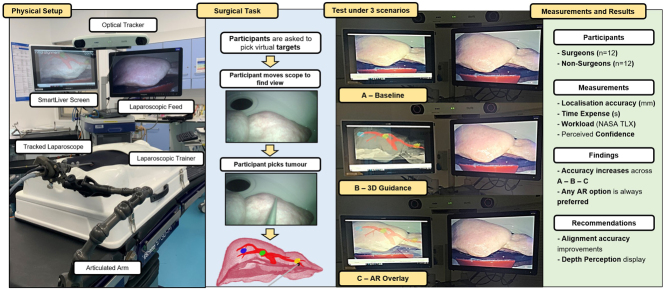

## 1 Introduction

The implementation of Augmented Reality (AR) has been widely suggested as a promising research avenue to minimise the risk of minimally invasive surgery (MIS) and therefore increase its uptake (Nicolau et al., 2011, Bernhardt et al., 2017). This is of particular importance for the case of laparoscopic liver surgery (Fuchs et al., 1998), where the reduced haptic feedback and limited freedom of instrument movement increases surgical risk when tumours are close to critical blood vessels (Wakabayashi et al., 2015). By overlaying a pre-operative model of the liver and internal vasculature on the laparoscopic video feed, surgeons can have more context on the relative location between the target tumour and major blood vessels. In recent years, the technical component of these systems that has received most attention has been the registration of the pre-operative model, i.e, the computational method through which the liver model is aligned to the laparoscopic video image (Labrunie et al., 2022, Koo et al., 2022). Even though multiple works present different approaches and extensive registration accuracy analyses (Prevost et al., 2020, Pelanis et al., 2021), there are no studies reporting the usability of the resulting overlay for the surgical task. In this paper, we present the first quantitative study on the usability of an AR overlay during laparoscopic liver surgery, using a phantom-based surgical environment.

## 2 Background

AR environments have been developed for surgical applications using optical see-through (OST) displays that project information on the optical field of view of the user (Birlo et al., 2022), video-see through (VST) displays where the information is projected over the video feed then presented on a screen (Cutolo et al., 2018), and autostereoscopic displays that project 3D information to the naked-eye, using either integral videography (Liao et al., 2004) or 3D autostereoscopic screens (Zhang et al., 2016). Autostereoscopy has been recently proposed to show an AR overlay in laparoscopic surgery (Zhang et al., 2022), but the need for a specific autostereoscopic screen and further image processing techniques to obtain the 3D display makes it currently costly for clinical translation. For laparoscopic surgery, OST displays are not usually considered as optical microscopes are not used and Head-Mounted Displays (HMD) (Birlo et al., 2022) are unsuitable as the surgeon must look at the laparoscopic video feed monitor. VST and OST HMD have also been combined in a single setup (Carbone et al., 2022), but VST with HMD is considered to be less effective for medical applications (Rolland and Fuchs, 2000). Therefore, AR overlays in MIS are usually deployed with a VST approach where information is overlaid on the endoscopic video feed and presented on either 2D (mono) or 3D (stereo) traditional stand up displays. Techniques to achieve this display have been reported for a variety of clinical applications, which include liver surgery (Prevost et al., 2020, Labrunie et al., 2022), kidney surgery (Teber et al., 2009), gastric surgery (Hayashi et al., 2016), hysterectomy (Collins et al., 2020), cranial base surgery (Mirota et al., 2011, Hussain et al., 2019) and prostate surgery (Simpfendörfer et al., 2011).

Typically, the main challenge in these approaches is to obtain an accurate alignment or registration between the pre-operative 3D structure of the organ requiring surgery and the laparoscopic video image of the organ at time of surgery. This problem is particularly difficult in the case of laparoscopic liver surgery, where large deformations between the pre-operative and intra-operative setting occur due to changes in patient positioning and the abdominal insufflation that is required for any laparoscopic procedure. Therefore, the main focus of this research field has been the development of methods that account for deformation (Pfeiffer et al., 2020, Labrunie et al., 2022) or methods that are potentially easier to employ clinically without any manual interaction by the surgeons (Robu et al., 2018, Koo et al., 2022).

Clinical translation studies on the use of AR in laparoscopic liver surgery have also mostly considered the registration step. Pelanis et al. (2021) and Luo et al. (2020) assessed registration accuracy and reproducibility in an animal setup. On clinical cases, Prevost et al. (2020) assessed not only registration accuracy, but also the resulting perceived AR overlay quality with a group of surgeons. In addition to these two factors, Schneider et al. (2020) further evaluated the feasibility of obtaining the registration manually or with a semi-automatic method during surgery. However, none of these studies have performed a formal usability study to assess the potential benefits of AR during a surgical task. An exception is an ex-vivo study presented by Adballah et al. (2022) where authors assess the improvement in tumour resection margins reduction of an AR overlay compared to conventional Laparoscopic Ultrasound (LUS) guidance. Despite quantifying surgical task performance, this study assessed an AR overlay that did not use any optical tracking system and was restricted to a single fixed laparoscopic view where the registration was performed. Therefore, the question of how a tracked AR overlay display affects a surgical task in the laparoscopic setting in terms of performance and usability still remains an open question. To date, studies that specifically consider usability and value questions on image guidance technologies in laparoscopic surgery have mostly focused on aspects such as video latency (Kumcu et al., 2017), laparoscopic video screen positioning (Van Veelen et al., 2002, Walczak et al., 2015) and use of 3D glasses (Lim et al., 2022).

In this paper, we present a novel study that evaluates the usability of an optically tracked AR display in laparoscopic liver surgery during the course of a simulated surgical task. Using a laparoscopic phantom setup, participants are asked to locate a set of virtual tumours while using an AR overlay provided by a previously reported system, the Smartliver system (Thompson et al., 2018, Schneider et al., 2020). Since this setup is not affected by intra-operative deformation, we are able not only to display an accurate overlay to the participants, but also to accurately measure the tumour localisation accuracy and therefore evaluate performance. Since optical tracking is used, participants are also allowed to move the laparoscope and explore the phantom surgical scene before the performing the localisation task, providing valuable information on how the AR overlay can provide surgical navigation. We test the simulated surgical task setup both with participants with an engineering background and with surgical background — for the surgical background group, we enforce ecological validity by performing the study in a real operating theatre. Additionally, we also consider two different AR display options, a conventional overlay where 3D information is superimposed to the video, and a separate option where the 3D information is detached from the video, resembling a surgical navigation scenario (as in the method of Hayashi et al. (2016)).

The main contributions of this paper include:

• The first study to assess how an AR overlay affects a surgical task in the laparoscopic environment in terms of performance and usability, using data from both surgeons and non-surgeons.
• A novel phantom study setup designed for the accurate measurement of surgical performance in a fairly realistic environment of AR guided laparoscopic liver surgery.
• A thorough comparison between two distinct image-guidance displays, providing insights on what is the most suitable AR visualisation to implement.

## 3 Materials and methods

In this section, we describe the study design through the following structure — in Section 3.1 we describe participant data, then in Section 3.2 we present our simulated surgery setup, in Section 3.3 we detail our study protocol and in Section 3.4 we finally describe our measurements and data collection.

### 3.1 Participant data

In this study, we considered two groups of 12 participants, one composed of clinical staff with experience in laparoscopic surgery, the “Surgeon group”, the other with an engineering background and no surgical experience that we refer to as “Engineering group”. In addition to the surgeons that represent the intended users of the tested AR system, we choose to include a group (Engineering group) without any prior surgical experience to act as a reference and provide general insights on the use of AR in the physical setup of laparoscopy.

In Table 1, we list not only the demographic data of these participants, but also their acquaintance with AR systems and years of experience in performing laparoscopic surgery. For simplicity, we separate the surgery experience in two groups, above 6 years which includes a range between 9 and 13 years of experience, and below or equal to 6 years which includes a range between 1 and 6 years of experience.

**Table 1:** Participant demographic data, experience with AR and experience with laparoscopic surgery.

	Groups	Engineering group	Surgeon group
Age	18–25	3	1
	26–30	5	3
	31–35	2	3
	36–40	0	3
	>40	2	2

Gender	Male	7	9
	Female	5	3

AR experience	Yes	5	1
	No	7	11

Laparoscopic surgery experience	≤6 years	**None**	5
	>6 years		7

**Fig. 1:**
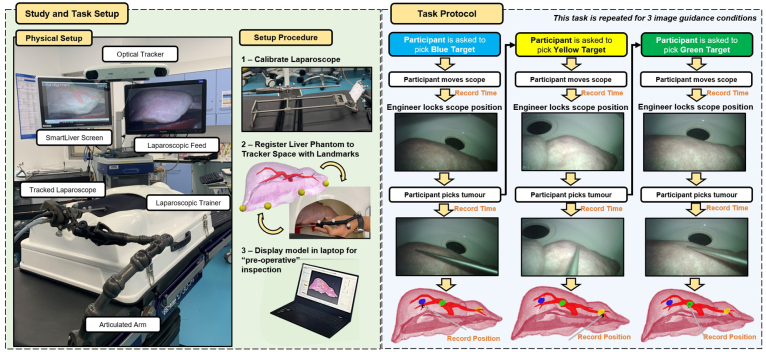
Study setup and protocol. In the left, “Physical Setup” shows the simulated AR guided surgery setup used in the operating theatres with surgeons, and respective components. In “Setup Procedure” are highlighted the preliminary steps performed by the study team before the surgical task: a calibration and liver phantom registration to enable AR guidance, and positioning of a display with the 3D model of the liver phantom. In the right is shown the sequential protocol for the localisation of liver phantom tumours. For each target tumour, the participant is allowed to freely move the laparoscope to obtain a view suitable for picking and then pick the tumour location using a tracked pointer in a fixed view. This process is repeated sequentially for the blue, yellow and green targets, and under three different AR conditions illustrated in Fig. 2. For every localisation, we record the laparoscope positioning time, tumour picking time and pointer position during picking.

### 3.2 Experimental setup

The setup used for the AR guided simulated surgical task is shown on the left side of Fig. 1. We test the Smartliver (Schneider et al., 2020) AR system for laparoscopic liver surgery, which comprises the following components:

• A laparoscopic system stack which includes a 3D stereo laparoscope and laparoscopic video screen (monitor on the right). Even though a stereo laparoscope is used, the AR system does not rely on any stereo features and only displays 2D images from one channel.
• An optical tracking system that comprises an infra-red camera positioned at the top of the stack, and a tracked marker that is attached to the laparoscope. Optical tracking enables the AR overlay to be projected from any laparoscopic view within the optical tracking system physical range, enabling surgical navigation on the scene.
• A calibration rig that is displayed in the “Setup Procedure” section of Fig. 1, and used to obtain a hand-eye calibration between the laparoscopic camera and the optically tracked marker, enabling the AR overlay to be accurately projected from an arbitrary view.
• A medical grade computer (left screen on the stack) with the Smartliver software, which is implemented on top of Scikit-Surgery libraries (Thompson et al., 2020).

In addition to the AR system, we set up a laparoscopic abdominal training box on top of a surgical table (Fig. 1), and position a silicon liver phantom with dimensions 290 × 118 × 126 mm provided by a commercial service^1^ inside in an anatomically realistic position with the aid of a 3D printed cast. The abdominal training box is covered with a neoprene sheet that has two holes, one for laparoscope insertion, and one for an optically tracked pointer, which is used to pick tumours in the localisation task. We position the laparoscope puncture access so that the video images capture the liver phantom with the same orientation as in real laparoscopic liver surgery. To stabilise the position of the laparoscope during tumour localisation, we attach its handle to the tip of an articulated arm that is anchored to the table and can be set to moving and locked states.

We run the study in two separate locations for the two different groups — the surgeon group tested the system in the operating theatres where they usually perform surgery, maximising ecological validity, and the engineering group performed tests in a university mock operating room whose purpose is image guidance technology development. Since the equipment tested in the engineering environment cannot be used in the operating theatres due to sterilisation requirements, the system had different components on each setup, which did not change functionality: the engineering group used a 3D Viking laparoscope^2^ and an NDI Polaris Vega^3^ tracker whereas the surgeon group used a Storz 3D laparoscope^4^ and an NDI Polaris Spectra tracker. The physical setup shown in Fig. 1 refers to the operating theatre environment tested by the surgeon group.

### 3.3 Protocol

#### 3.3.1 AR system setup

The first step of our protocol is to set up the Smartliver guidance system, as illustrated under “Setup Procedure” in Fig. 1. Using the Smartliver user interface, we first calibrate the laparoscope to the optical tracking coordinate system using a method based on a clinically compatible calibration rig that has been previously tested in Dowrick et al. (2023) and evaluated in terms of usability in van Berkel et al. (2020). Once the laparoscope is calibrated, we register the 3D model of the liver phantom to the optical tracking space by physically picking the position of 4 pre-defined anatomically distinctive landmarks in the phantom edges with an optically tracked pointer and performing a point-based registration (Arun et al., 1987). The 3D model of the phantom is then projected onto the laparoscopic view by multiplying the obtained registration by the hand-eye calibration and the laparoscope optical tracked information. Clinically, a rigid registration based on landmarks would not be guaranteed to be feasible nor sufficiently accurate in laparoscopic surgery, and an automatic method such as the ones proposed by Labrunie et al. (2022) and Koo et al. (2022) would be necessary. However, since the aim of this study is to purely assess the benefit of AR with a quantitative evaluation, we choose to use a deformation free environment with a simple rigid registration. Finally, we position a laptop in front of the abdominal training box with an interactive display (Clarkson et al., 2015) of the 3D model of the liver phantom which includes liver surface, blood vessels, and the virtual target tumours. To ensure that our further localisation error measurements are valid, we only proceed to the surgical task if the landmark-based registration has a mean fiducial registration error below 3 mm.

#### 3.3.2 Tumour localisation task

**Fig. 2:**
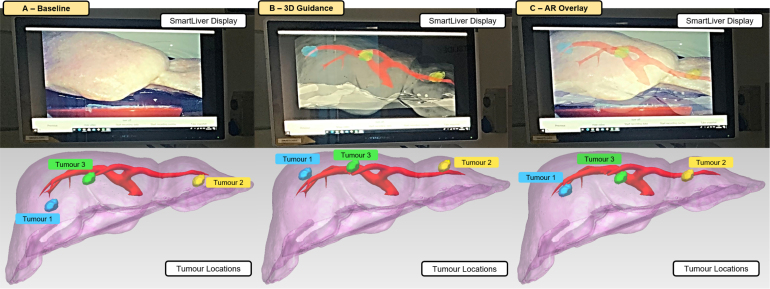
AR guidance tested conditions and respective tumour targets. Top shows real photos of the Smartliver computer side-by-side with the laparoscopic video feed. The bottom shows the respective target tumour locations inside the liver phantom for each condition: Tumour 1 is represented with blue, Tumour 2 with yellow and Tumour 3 with green. These are the colours that are displayed during the picking task (except for the baseline condition).

Once the Smartliver system is setup, we first position the laparoscope in a view where the liver is not visible as a starting point. Then, we introduce the participant to the concepts of AR and laparoscopic surgery, and briefly explain the tumour localisation task described on the right side of Fig. 1. The objective of this task is to localise, intra-operatively, in the physical liver phantom, a set of three artificially created virtual tumours that are shown in the pre-operative 3D display in the laptop, and also on the Smartliver AR guidance system. These virtual tumours are identical in shape and size with an approximate diameter of 20 mm, and are uniquely identified by distinct colours and representative anatomical locations in the liver (details in Fig. 2): the first tumour is highlighted with blue and located in the right lobe, the second tumour is highlighted in yellow and located in the left lobe and the third tumour is highlighted in green and located in the right lobe but closer to the falciform ligament.

In order to faithfully represent the surgical scenario, before the localisation task we instruct the participant to interact with the 3D surface model displayed in the laptop and inspect the position of the target tumours without any time limit. This inspection step aims to simulate the surgical planning stage where surgeons analyse a pre-operative scan of the liver to be operated, and mentally map the location of the tumours to be resected and its geometrical relation to relevant surface and vascular landmarks. Once the pre-operative inspection is complete, we ask the participant to locate the blue tumour following the routine illustrated in right side of Fig. 1:

• Firstly, we unlock the articulated arm holding the laparoscope, and ask the participant to move until a view from which the tumour location can be pinpointed is found.
• We then lock the articulated arm on the selected view, and ask the participant to use a tracked pointer to touch on the liver phantom surface point they believe to be closest to the tumour.
• Once the participant is satisfied with the pinpointed surface location, we record the pointer position, and repeat the protocol for the yellow and green tumours sequentially.

**Fig. 3:**
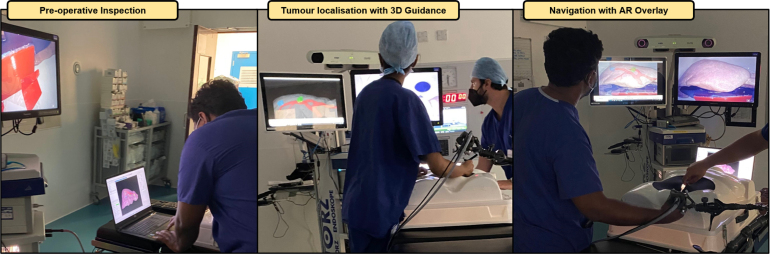
Examples of different stages of the simulated surgical task being undertaken by the surgeon group. Left shows the pre-operative inspection step, middle shows a participant performing tumour localisation using the 3D guidance condition, and right shows a participant navigating the laparoscope with the AR overlay condition.

By separating the task into view selection and static view tumour picking, we allow the participant to not only perform a targeting task with AR, but also to navigate the laparoscopic scene. We specifically fix the order of picked tumours to be blue (right lobe) → yellow (left lobe) → green (centre) in order to encourage participants to always perform the navigation step before tumour picking — if we transitioned from the blue to the green tumours, the participant could easily use the same laparoscopic view. Since the tracked pointer used for localisation does not pierce the liver phantom tissue, we specifically position the virtual tumours close to the anterior surface to minimise the effect of tumour depth in the resulting pinpointing accuracy measurement. The surgical task is repeated under three different AR guidance scenarios or conditions, which we describe in the next subsection. Examples of surgeons performing different steps of this localisation task are shown in Fig. 3.

#### 3.3.3 AR guidance conditions

In order to be able to provide a comparative analysis on how AR can affect usability and performance in a surgical task, we define three image guidance conditions that differ on the content that the Smartliver interface displays and are represented in the top row of Fig. 2:

• **A-Baseline**: no overlay is provided, simulating the real surgery scenario where surgeons mainly use their mental mapping of pre-operative information to make decisions. This condition sets a performance baseline for the task.
• **B-3D Guidance**: the 3D model is aligned to the laparoscopic view, but instead of being overlaid to the real laparoscopic video, it is superimposed on a black screen, simulating a more conventional surgical navigation display as tested by Hayashi et al. (2016).
• **C-AR Overlay**: the 3D model is aligned and superimposed on the laparoscopic video, as in the typical application of AR.

We consider the 3D Guidance condition B, because as well as evaluating the value of AR, we also aim to investigate whether the direct overlay of the aligned 3D model onto the laparoscopic video is the most suitable AR display for image guided surgery.

Each participant repeats the localisation task previously described in 3.3.2 under these three different conditions. In order to prevent the participants from learning the tumour locations across conditions, we create three separate sets of virtual tumours, as shown in the bottom of Fig. 2 — the anatomical regions and colours of the targets are kept the same, but with slight variations in position. Additionally, to further reduce learning bias across tasks in further data analysis, the ordering is randomised so that there are 2 participants testing each of the 6 permutations of ordering A, B and C.

### 3.4 Data collection

We collect quantitative data during two stages of the study, during the task and after task completion. During the task, for each tumour and condition, we record the time taken to find an optimal view, the time taken to perform the tumour picking and the position of the pointer during picking. With the tracked pointer position, we measure picking accuracy as the Euclidean distance between the pointer tip and the closest virtual tumour surface point after applying the landmark-based registration result obtained during setup to the tumour coordinates. We choose to not use the tumour centroid to minimise the contribution of tumour depth to the localisation error, and consider the chosen measurement to be sufficient for a comparative study. After each condition, participants were first asked to quantify how confident they were in their localisation decision for each tumour, in a scale of 1 to 7. Then, participants rated the overall condition usability and workload by filling out NASA Task Load Index (TLX) (Hart and Staveland, 1988) questionnaires, which included scales for Mental, Physical and Temporal demands, Effort, overall Performance and Frustration level. Finally, participants provided subjective feedback on their overall experience, which includes the preferred condition, limitations, and design improvements.

**Fig. 4:**
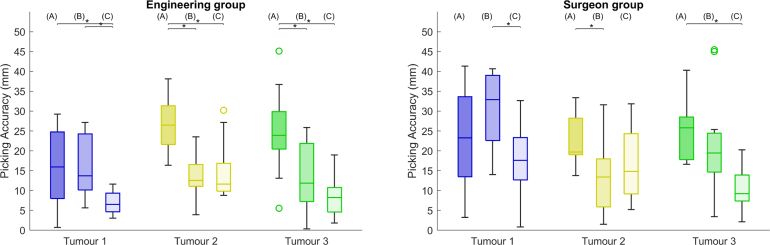
Virtual tumour localisation accuracy for three distinct tumours across three different image guidance conditions, in mm. (A) refers to the baseline, (B) to 3D Guidance and (C) to the AR Overlay. Lower values represent lower localisation errors, and therefore higher accuracy. Left and right show the engineering and surgeon group measurements, respectively. In each tumour analysis, black lines and markers indicates statistically significant pair-wise comparison with p-value < 0.05, and circles represent outliers.

**Fig. 5:**
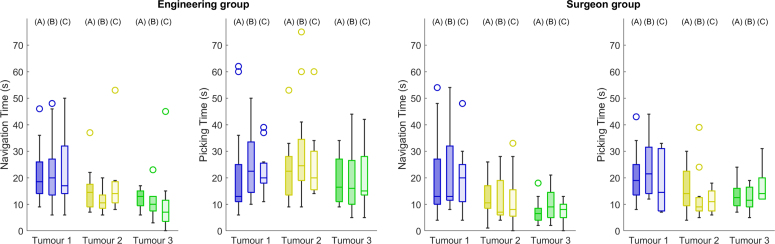
Time spent during navigation and localisation for three distinct tumours across three different AR conditions, in seconds. (A) refers to the baseline, (B) to 3D Guidance and (C) to the AR Overlay. Left and right show engineering and surgeon group measurements, respectively. Within each group, left shows navigation time and right the picking time. Black lines and markers indicates statistically significant pair-wise comparison with p-value < 0.05, and circles represent outliers.

## 4 Results

In this section, we present all of our quantitative data in boxplots with median and Interquartile Range (IQR) separately for each tumour target, image guidance condition and participant group. To test the hypothesis that the three conditions A, B, C lead to significantly different results, we perform Friedman tests with multiple comparisons for each tumour picking within each participant group, and use a Bonferroni correction with α = 0.05 to evaluate pairwise comparisons. We choose this test as we assume the tumour picking measurements under different conditions to be repetitions from the same individual that do not follow a Gaussian distribution.

### 4.1 Localisation accuracy

Tumour localisation accuracy results for the engineering and surgeon groups across three conditions are presented in mm for each target tumour in Fig. 4. For the engineering group, we observe that the AR overlay option significantly increases the pinpointing accuracy compared to the baseline, with Tumour 1 errors decreasing from 15.9 [8.0; 24.7] mm to 6.5 [4.6; 10.7] mm, Tumour 2 errors decreasing from 26.5 [21.6; 31.3] mm to 11.6 [9.8; 16.8] mm and Tumour 3 decreasing from 23.9 [20.5; 29.9] mm to 8.2 [4.6; 10.7] mm. The 3D guidance condition also improves the accuracy compared to the baseline, but not significantly for Tumour 1 — in this case the overlay significantly improves accuracy over the 3D guidance condition. Overall, median values show that accuracy performance increases in the trend A → B → C.

For the surgeon group, significant improvements relative to the baseline are observed for the AR overlay condition in Tumour 3 with an improvement from 25.8 [17.8; 28.5] mm to 9.2 [7.3; 13.9] mm, and for the 3D guidance condition in Tumour 2 with an improvement from 19.7 [19.0: 28.2] mm to 13.4 [5.9: 17.9] mm. For Tumour 1, a significant improvement is observed from the 3D Guidance to the AR overlay conditions. Compared to the median trend observed in the engineering group, the 3D Guidance system does not increase the accuracy of Tumour 1 localisation over the baseline, and Tumour 2 localisation is slightly less accurate with the AR overlay than with the 3D guidance display. Apart from an exception in Tumour 1, either methods of image guidance displays lead to lower median localisation errors compared to the baseline.

### 4.2 Time expense and perceived confidence

Results for the measured time expenses during the surgical task are presented in seconds for both groups in Fig. 5. As mentioned in the protocol Section 3.3, we consider two time measurements, the time spent in finding an optimal laparoscopic view for picking (Navigation Time) and the time taken to pick a phantom surface location with the pointer (Picking Time). For both measurements and participant groups, no statistically significant differences are observed across the three conditions for each tumour target. Differences are mainly observed in median values across different tumours and participant groups — Tumour 1 shows higher values than Tumours 2 and 3 except in the picking time of the engineering group, and surgeons spent less time than the engineering group in picking Tumours 2 (decrease from 22.5, 24.5, 20.0 to 14.0, 9.0, 7.5 for A, B and C respectively) and 3 (decrease from 16.5, 16.0, 15.0 to 12.5, 11.5, 14.0 for A, B and C respectively).

**Fig. 6:**
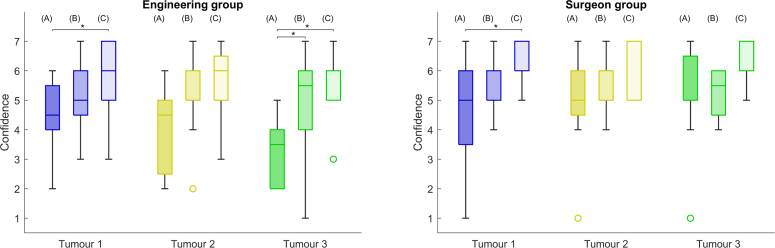
Perceived confidence values during localisation of three distinct tumours across three different AR conditions, in a scale of 1 to 7. (A) refers to the baseline, (B) to 3D Guidance and (C) to the AR Overlay. Black lines and markers indicates statistically significant pair-wise comparison with p-value < 0.05, and circles represent outliers.

Results for the perceived confidence in tumour localisation are presented in Fig. 6. We observe that the AR overlay display significantly increases the confidence of the engineering group over the baseline for Tumour 1 with an increase from 4.5 [4.0; 5.5] to 6.0 [5.0; 7.0], and for Tumour 3 with an increase from 3.5 [2.5; 4.0] to 6.0 [5.0; 6.0]. Similarly to the accuracy results of Fig. 4, median values follow an increasing trend across the 3 conditions, with the 3D guidance condition only introducing a significant confidence increase over the baseline for Tumour 3 from 3.5 [2.5; 4.0] to 5.5 [4.0; 6.0].

**Fig. 7:**
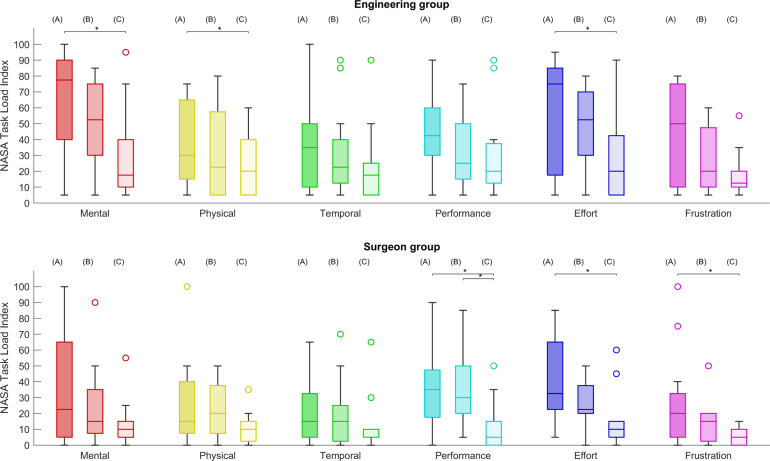
NASA TLX scale values reported for three different AR conditions and two participant groups. (A) refers to the baseline, (B) to 3D Guidance and (C) to the AR Overlay. Black lines and markers indicates statistically significant pair-wise comparison with p-value < 0.05, and circles represent outliers.

Confidence results for the surgeon group follow a similar median trend to the engineering group, but with overall higher values. For all tumours, surgeons reported highest confidence values for the AR overlay option and lowest for the baseline, with Tumour 1 showing an increase from 5.0 [3.5; 6.0] to 7.0 [6.0; 7.0], Tumour 2 an increase from 5.0 [4.5; 6.0] to 7.0 [5.0; 7.0] and Tumour 3 an increase from 5.0 [3.5; 6.0] to 7.0 [6.0; 7.0] — however, only Tumour 1 shows a statistically significant difference. Confidence values obtained with the 3D Guidance condition show median values in between the baseline and AR overlay conditions, but do not show any significant pair-wise differences.

### 4.3 Usability and workload

NASA TLX values filled in by the participants are summarised in Fig. 7. For both groups the median values of almost all the workload factors decrease with the trend of A → B → C, suggesting that the AR overlay leads to the lowest workload and the baseline the highest — a single exception is observed in the physical demand experienced by the surgeon group, where a slight increase is observed from A to B. Significant improvements over the baseline are observed in the engineering group for the mental demand, physical demand and effort when using the AR overlay display, with respective workload decreases from 77.5 [40.0; 90.0] to 17.5 [10.0; 40.0], 35.0 [10.0; 50.0] to 17.5 [5.0; 25.0] and 75.0 [17.5; 85.0] to 20.0 [; 40]. The 3D guidance display shows workload reductions compared to the baseline, but without significant pair-wise comparisons. For the surgeon group, significant comparisons are observed in the overall performance, effort and frustration level scales. In these cases, the AR overlay leads to a significantly lower workload than the baseline, with performance scale decreasing from 35.0 [17.5; 47.5] to 5.0 [0.0; 15.0], effort decreasing from 32.5 [22.5; 65.0] to 10.0 [5.0; 15.0] and frustration decreasing from 20.0 [5.0; 32.5] to 5.0 [0.0; 10.0]. A significant decrease in overall performance is also observed from the 3D guidance to AR overlay conditions. Overall, all median values suggest that compared to the engineering group, the surgeons found all conditions to have a lower workload and higher usability.

### 4.4 Subjective assessment

Subjective feedback from both participant groups indicates that either of the image guidance conditions are always preferred over the baseline. Between the two tested displays, the AR overlay was preferred by 15 participants (6 in the engineering group and 9 in the surgeon group), 3D guidance was preferred by 7 participants (5 in the engineering group and 2 in the surgeon group) and 1 participant from each group did not have a preference. Overall, participants indicate that the 3D information display provided them with an improved perception on the overall location of the liver, blood vessel, and target tumours in the laparoscopic view. To understand the differences in preference, we highlight the main limitations and comments raised by participants:

• **Spatial awareness**: All participants highlighted that image guidance provided a better geometrical context on the position of the liver, blood vessels and target tumour. However, the AR overlay was always considered to be the best at integrating information as it matched directly the pre-operative anatomy onto the intra-operative laparoscopic video. Additionally, surgeons highlighted that the AR overlay could improve resection margin (3 surgeons) and lead to fewer mistakes (1 surgeon).
• **Pointer visualisation**: In addition to the matching anatomy, many participants highlighted the fact that visualising the tracked pointer overlaid on the pre-operative data helped the localisation task. Specifically, for 3 surgeons and 1 engineering participant, the inability to see the tracked pointer in the 3D guidance black screen made this option less preferable. Conversely, 3 engineering participants considered that the overlay of the video with pointer and AR overlay was too cluttered, and therefore preferred the 3D guidance option.
• **Overlay quality**: 2 surgeons and 5 engineering participants considered the AR display to not be accurate enough in the AR overlay option. For a smaller set of participants, this factor was decisive 3D guidance as they found the overlay inaccuracy to be misleading, 2 engineering participants preferred the AR overlay option regardless as they felt they could correct for the overlay inaccuracy visually observing the silhouette of the real liver phantom.
• **Video Latency**: 3 surgeons and 1 engineering participant found the AR overlay display to have a small time lag compared to the real video, which led to a slight discomfort during visualisation. However, only 2 of these participants found this issue relevant enough to prefer the 3D guidance option.

For both AR options, a final concern raised by the participants was the lack of depth perception in the overlay visualisation (2 surgeons and 1 engineering participant). These participants mentioned that both AR options would need improvements in depth perception visualisation in order to locate or operate on tumours located deeper in the liver (surgeons), and discern the depth of overlapping blood vessels and tumours (engineering participant).

## 5 Discussion

In this section, we use the presented results to discuss the value of AR in performance and usability, and potential requirements necessary for the application of this technology to laparoscopic surgery.

### 5.1 Performance

Overall, tumour localisation accuracy results from Fig. 4 suggest that either guidance displays (AR overlay or 3D guidance) displays improve spatial perception of the laparoscopic scene, and have the potential to improve surgical task performance. Even though median accuracy results from the engineering group point to an increase in performance across conditions (A) Baseline → (B) 3D Guidance → (C) AR Overlay, this is not clearly evident for the distributions of each target tumour, mainly in the surgeon group. In fact, this trend is only clearly observed in the accuracy results of Tumour 3: for Tumour 1 there is not a significant improvement when using 3D guidance versus the baseline; for Tumour 2, both AR options surpass the baseline, but without an obvious separation between them. After retrospective analysis of the feedback of the participants on their perceived confidence in the localisation task, we hypothesise that these differences towards the A → B → C trend are explained by the inherent difficulty in pinpointing different tumours across conditions.

**Fig. 8:**
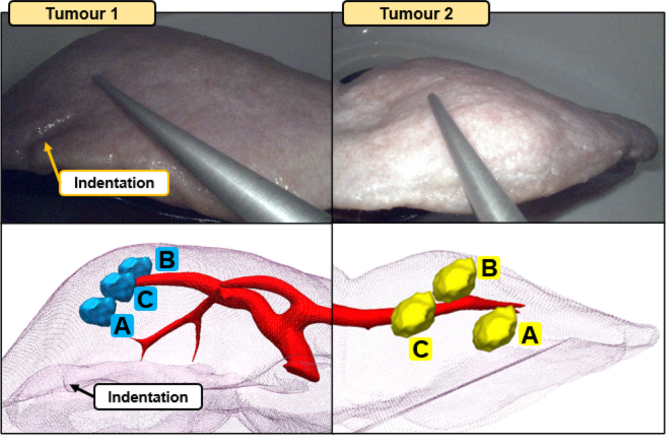
Tumour 1 and Tumour 2 locations across three different image guidance conditions captured from a representative laparoscopic view. Top shows a captured view from a surgeon participant picking a target tumour under the 3D guidance condition B, bottom shows a roughly aligned view of the pre-operative model with all the virtual tumours. This is a retrospective analysis view that was not displayed to participants during the study.

**Fig. 9:**
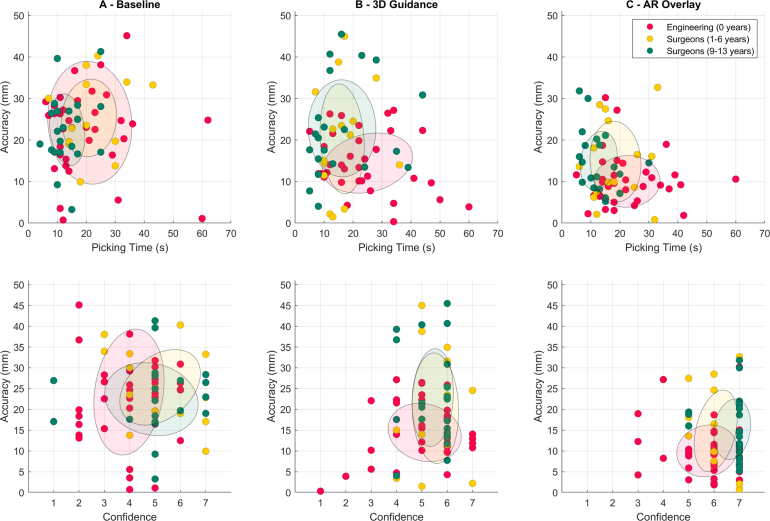
Distributions of accuracy and tumour picking time (Top) and accuracy and perceived confidence (Bottom) for three different levels of surgical experience across three image guidance conditions. The engineering group is considered as the reference with 0 years of surgical experience. Each point corresponds to a single tumour localisation, and each participant contains 3 points in each chart. For a qualitative comparison across conditions, a Gaussian fit is presented to assess distribution differences.

To further explain this difficulty variability, Fig. 8 shows the location of Tumours 1 and 2 for all three conditions from a representative laparoscopic view that participants used in this study. On the left where the locations of Tumour 1 are displayed, we can see that the location of the baseline condition A is fairly close to a liver surface indentation that is visible in the pre-operative model, whereas the location of the 3D guidance condition B is more posterior and further away across the camera depth direction. This configuration explains the lack of improvement from condition A to B in Tumour 1 — the display of 3D guidance display was not helpful enough to make the localisation of the tumour as easy as in the baseline, where a clear liver surface feature is present. This observation is in agreement with the feedback from all participants, as they mentioned the surface indentation as an anatomical reference. A similar analysis can be performed for Tumour 2 whose locations are presented on the right side of Fig. 8. In this case, the tumours are located in the smaller left lobe where many liver edges and anatomical cues are visible. However, for condition B, the tumour is more superficial and easier to pinpoint in the laparoscopic view than for condition C, explaining the similar accuracy distributions observed in Fig. 4. For Tumour 3, all locations were similarly difficult as there were no anatomical cues to use (hence the lower confidence values obtained in Fig. 6). Therefore, results suggest that AR is mostly valuable when fewer cues are available, and that the performance trend A → B → C might be observed if all tumours had the same level of difficulty.

Navigation and picking times of Fig. 5 indicate that none of the tested displays reduced the time expense of the task. Higher navigation times were observed for Tumour 1, possibly due to the fact that this was always the first target to be picked where participants were getting acquainted with the image guidance display. From our observation, despite providing more context on the surgical scene, the AR display needed to be interpreted, and therefore time expense was not particularly reduced. However, this does not mean that AR will not reduce operating time during live surgery. During our study, participants were always allowed to inspect the liver phantom model in a laptop (see Fig. 1), and we counted the number of participants that needed to re-inspect the model during the localisation task: 10 for the baseline, 3 for 3D guidance and 2 for the AR overlay. These observations support the idea that AR can potentially lead to quicker decision making.

### 5.2 Effect of surgical experience

Even though time measurements do not show significant differences across conditions, median values differ across the two groups, with the surgeons spending less time during picking. Additionally, the surgeon group obtains overall lower accuracy values (higher errors) than the engineering group. To better understand these differences, we further separate the surgeon group in two sub-groups according to their years of experience in laparoscopic surgery (as in Table 1), and present point distributions of accuracy and respective picking time for the three tested conditions in the top of Fig. 9. In these charts, we consider the engineering group as the reference with 0 years of experience in surgery, and refer to the distribution centroids (highlighted with a transparent Gaussian fit) for analysis. In these distributions it is clear that the least experienced group (engineering with red colour) spends the most time during picking across the three conditions, and increases accuracy across them (AR overlay with the highest and baseline with the lowest). A similar effect is observed for the less experienced surgeons (yellow) where accuracy does not improve as much as for the engineering group, but less time is spent as well. For the most experienced surgeons (green), picking times are the lowest, and accuracy improvements are less pronounced across the three conditions. These results suggest that the least experienced users are the ones that benefit the most from the AR display. From our observations during the study and subjective feedback, we hypothesise that surgical task performance is affected by previous anatomical knowledge of the individuals. When the most experienced surgeons are presented with the pre-operative model of the liver phantom, they relate their inspection to the real segmental anatomy of the liver (Couinaud, 1999), and perform the localisation task quickly with a coarse precision. Conversely, the engineering group participants do not use any prior anatomical knowledge during their task — instead, they consider the liver to be a generic 3D object, and try to pinpoint the location of the target tumours with maximal precision, at the cost of more time. The less experienced surgeons show a behaviour between the two previous groups as they have less confidence in their anatomical knowledge of the liver than the most experienced surgeons, and perform the surgical decision more conservatively. Charts with distributions of accuracy and corresponding perceived confidence presented in the bottom of Fig. 9 support this hypothesis — perceived confidence only increases substantially across conditions for the group without surgical experience nor anatomical knowledge. Between the two surgeon groups, confidence is higher for the expert surgeons, but a higher increase in accuracy is observed for the less experienced. Overall, our results indicate that AR could be of value especially for individuals undergoing training, by helping them on decision making and increasing their confidence.

**Fig. 10:**
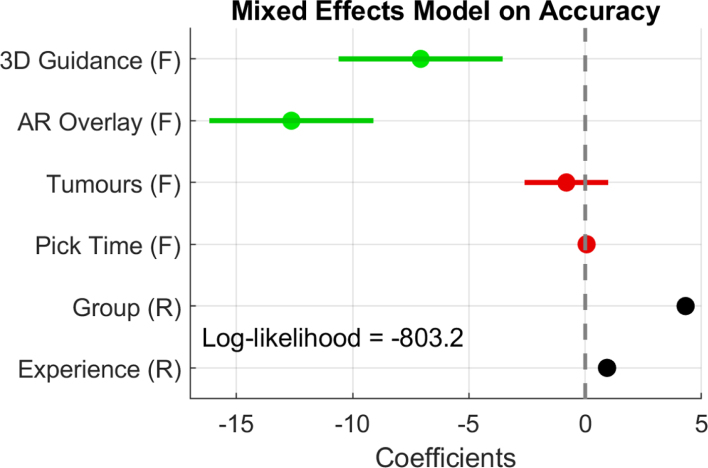
Mixed effect model fit on localisation accuracy. Fixed effects are highlighted with (F) and Random effects with (R). Significant fixed effect coefficients with p-value < 0.05 are highlighted in green, whereas non-significant are presented in red.

In order to provide a final robust statistical result on the effect of the AR displays on the performance, we fit a Mixed Effect Model (Bishop and Nasrabadi, 2006) to our data considering the Baseline condition A as a reference and localisation accuracy as the response. As fixed effects we consider the 3D Guidance and AR Overlay conditions separately, the different tumours and the picking times. As random effects we consider the participant group as the intercept, and the experience subgroups as the slope. In total, we consider a sample of 216 values (24 participants with 9 measurements each), and obtain the model coefficients and confidence intervals presented in Fig. 10. Significance is observed for the two guidance conditions with negative coefficient values, meaning that they influence the localisation error negatively, and increase the accuracy. As expected, the AR overlay obtains the coefficient with the highest absolute value, being the effect that increases accuracy the most. Compared to these conditions, the tumour location and picking times do not present significant effects on accuracy. In agreement with previous analyses, the participant group random effect shows a positive coefficient, indicating that there is an increase in the localisation error from the engineering to the surgeon groups.

### 5.3 Usability

NASA TLX values of Fig. 7 indicate that task workload decreases with the same trend as performance increases, with the AR overlay showing the lowest workload and the baseline the highest. Regarding specific scales, only the engineering group reported significant reductions in mental and physical demands. This may be explained by the acquaintance of surgeons with the laparoscopic setup, as it is not intuitive for inexperienced individuals to manipulate a laparoscope within the abdominal cavity while looking at a screen. Interestingly, only surgeons report significant overall performance improvements. This result agrees with the perceived confidence results, where surgeons report much higher values when using the AR overlay option.

Between the two guidance options, the AR overlay leads to all but one of the statistically significant improvements, indicating that this option is superior to 3D guidance not only in performance, but also usability. Feedback from both groups suggest that the 3D guidance option is less useable as it separates the view in two screens, forcing a user to mentally align the 3D model view with the laparoscopic video, increasing the workload. Therefore, even though separating the 3D model display from the video seems appealing as it reduces confusion in the surgical scene, conventional AR superimposition on video leads to superior performance and usability results.

### 5.4 System requirements and future considerations

Subjective feedback from all participants was that AR has potential benefits in surgery. Between the two tested displays, it is clear that the overlay is the most preferred way of image guidance, mainly in the surgeon group. Interestingly, the 3D guidance display was mostly preferred due to issues experienced with the AR overlay such as video lag and alignment inaccuracy. In fact, only 3 engineering participants preferred the 3D guidance over the AR overlay in terms of design due to its less “cluttered” view — however, these participants still felt more confident when using the overlay. Using this data, we were able to compile a set of system requirements that should be taken into account in the design of AR solutions in laparoscopic surgery.

Firstly, current ongoing research in improving registration and tracker calibration accuracy should be continued in order to improve overlay quality in the real clinical scenario. In this study, we tested a tracked AR laparoscopic system with representative sources of calibration and tracking errors, and observed that even under a rigid environment, the overlay can show a visible misalignment from differently positioned laparoscopic views. This projection error is expected, and potentially explained by the “lever-arm” effect (Xiao et al., 2018) that occurs when calibrating long scopes in which the optical marker is positioned far from the camera. To mitigate this effect and improve the visualisation, registration algorithms should be fast enough to perform a repetition or simply a refinement once a stable view of interest is found — a certain level of inaccuracy may be tolerable when navigating the scene, but within a view of interest where tumour resection margins are evaluated the display error should be minimised as much as possible. Ideally, a deformable registration such as the one by Labrunie et al. (2022) should be employed at this stage to increase the accuracy of the display. However, validation of these algorithms is still difficult, and other sources of intra-operative imaging such as Laparoscopic Ultrasound (LUS) would have to be used to verify tumour locations. If the visual inspection of LUS images is not enough for verification, these could be aligned either to the camera view (Rabbani et al., 2022, Liu et al., 2021), or to the deformed pre-operative CT (Ramalhinho et al., 2020, Ramalhinho et al., 2022). Regardless of how accurate the display is at any arbitrary view, the silhouette of the 3D liver surface should be highlighted with a distinguishable colour, allowing the user to visually perceive how misaligned the model is relative to the video, and make a better informed decision. Additionally, live segmentation results of liver edges could be also displayed in colour to provide a direct visualisation on the difference between intra and pre-operative anatomy. Overall, improving the interpretation of the validity of the overlay is essential as in this study some participants obtained positioning errors above 30 mm that are not acceptable for clinical translation.

Secondly, in terms of hardware, improving video streaming from the laparoscopic system to the AR display computer is also crucial, as the perceived temporal lag between screens can affect overall usability. Thirdly, the main pitfall of the 3D guidance option was the inability to visualise the pointer — this display could still be explored if a real time virtual display of the pointer position (or any laparoscopic tool) was added. Finally, integration of depth information in the 3D model for either guidance display is desirable, using for example different colouring or opacity parameters (Reissis et al., 2023).

### 5.5 Study limitations

Even though we attempted to reproduce the surgical setting as closely as possible, our study design had some limitations. Since the pointer could not pierce the liver phantom, we designed tumour locations that were superficial and not exactly representative of the surgical scenario where AR is relevant. Future studies should consider how to perform localisation of deeper lesions, and further include an evaluation on how AR influences depth perception. Additionally, the tumour locations per condition and order were fixed. For a comparative study, we believe that these factors did not introduce a bias, even though we observed retrospectively that the location of the blue Tumour in condition B was more difficult than the others. In the future, randomisation of target location and order should be considered. Our study did not address how the 3D display of blood vessels affects the AR guidance — this could be explored further by designing a more complex vascular tree and test different visualisation options (Preim and Oeltze, 2008). A final relevant drawback of this study is the lack of intra-operative deformation. However, the presented study design could be easily adapted to account for this aspect by considering an AR overlay with a deformed 3D model of the phantom. In this case, guidance would be provided with a pre-operative surface that does not match exactly the intra-operative one, as expected in surgery.

## 6 Conclusion

In this paper, we have presented a phantom study to assess the value of AR in laparoscopic surgery. Using a novel phantom-based surgical environment, we designed a virtual tumour localisation task where the performance of 24 participants (12 surgeons and 12 non-surgeons) was objectively measured under three distinct image guidance conditions: conventional surgery without guidance, a display with the pre-operative 3D model aligned to the laparoscopic view on a black screen, and a conventional AR overlay. Results suggest that either guidance displays bring improvements in performance and usability compared with conventional surgery, with the single screen option showing the most significant results. We also observed that AR can have more impact in surgeons with less experience and knowledge of the operated anatomy. For future application, almost all participants prefer the single screen AR overlay as well, but believe that registration accuracy should be improved and that depth perception should be further included in the display. Future work will consider the use of the proposed setup for both training and skill assessment of surgeons in AR guided surgical environments, and for testing AR displays in the presence of intra-operative deformations.

## Declaration of competing interest

The authors declare that they have no known competing financial interests or personal relationships that could have appeared to influence the work reported in this paper.

## Data Availability

Data will be made available on request

## Appendix A Supplementary data

Video S1 3D surface of liver phantom and location of virtual target tumours.

## Footnotes

1 https://www.healthcuts.com/.

2 http://www.conmed.com/.

3 http://www.ndi.com/.

4 http://www.storz.com/.
